# Intraoperative visualization of nerves using a myelin protein-zero specific fluorescent tracer 

**DOI:** 10.1186/s13550-021-00792-9

**Published:** 2021-05-29

**Authors:** Tessa Buckle, Albertus. W. Hensbergen, Danny M. van Willigen, Frank Bosse, Kevin Bauwens, Rob C. M. Pelger, Fijs W. B. van Leeuwen

**Affiliations:** 1 Interventional Molecular Imaging Laboratory, Department of Radiology, Leiden University Medical Center, Albinusdreef 2, 2300 RC Leiden, The Netherlands; 2 Neurologische Klinik, Heinrich-Heine University Dusseldorf, Düsseldorf, Germany; 3 ORSI Academy, Melle, Belgium; 4 Department of Urology, Leiden University Medical Center, Leiden, The Netherlands

**Keywords:** Nerve imaging, Fluorescence-guided surgery, Myelin protein zero, Targeted imaging

## Abstract

**Background:**

Surgically induced nerve damage is a common but debilitating side effect in oncological surgery. With the aim to use fluorescence guidance to enable nerve-sparing interventions in future surgery, a fluorescent tracer was developed that specifically targets myelin protein zero (P0).

**Results:**

Truncated homotypic P0 protein-based peptide sequences were C-terminally functionalized with the far-red cyanine dye Cy5. The lead compound **Cy5-P0**_**101–125**_ was selected after initial solubility, (photo)physical and in vitro evaluation (including P0-blocking experiments). **Cy5-P0**_**101–125**_ (*K*_D_ = 105 ± 17 nM) allowed in vitro and ex vivo P0-related staining. Furthermore, **Cy5-P0**_**101–125**_  enabled in vivo fluorescence imaging of the Sciatic nerve in mice after local intravenous (i.v.) administration and showed compatibility with a clinical fluorescence laparoscope during evaluation in a porcine model undergoing robot-assisted surgery. Biodistribution data revealed that i.v. administered **[**^**111**^**In]In-DTPA-P0**_**101–125**_ does not enter the central nervous system (CNS).

**Conclusion:**

**P0**_**101–125**_ has proven to be a potent nerve-specific agent that is able to target P0/myelin under in vitro, ex vivo, and in vivo conditions without posing a threat for CNS-related toxicity.

**Supplementary Information:**

The online version contains supplementary material available at 10.1186/s13550-021-00792-9.

## Background

Surgery, and science therein, has come a long way since Mr. Gunning and Lord Thurlow, respectively, stated to each other in 1796: “there is no more science in surgery”, in reply “than there is in butchery” [1]. Contradictory to these statements, the rapid translation of innovative minimally invasive surgical technologies has initiated the concept of “precision surgery” (2–5). This concept is mostly driven by engineering efforts in the form of medical devices such as endoscopic cameras, refined instruments, and robotic manipulators that enable modern surgeons to intervene in the human body in ways that were previously not thought possible [3, 4, 6]. Imaging provides an alternative to impact surgical accuracy; the application of minimally invasive procedures is strengthened by the ability to map areas of disease in the context of healthy anatomy (so-called surgical roadmaps) using non-invasive preoperative imaging modalities such as MRI or PET/CT [7–9]. Unfortunately, intraoperative detection of preoperatively identified lesions/structures can be challenging. For instance, increased distancing between the surgeon and the patient limits the surgeon’s sensory experience in the form of palpation (e.g. when a surgical robot is used). This shortcoming can at least in part be compensated through the use of interventional molecular imaging. To date, this imaging sub-discipline has predominantly focused on intraoperative detection of cancerous lesions using either radio- or fluorescence guidance [10, 11]. Here the main applications have included complex anatomies such as the head-and-neck or pelvic area where image guidance is exploited for both detection of nodal metastases and primary tumour margins [12–16]. However, in these same anatomies accidental surgical damage to nerves can yield debilitating side effects such as loss of sensory feeling or speech, incontinence and/or erectile dysfunction. This occurrence of surgically induced nerve damage is not uncommon: despite the fact that more than 70% of prostate cancer patients receive nerve-sparing surgery, it is accepted that 30% of patients suffer from loss of erectile function at 1 year post-surgery [17]. Here, it should be noted that the extent of the damage may be hard to predict [18]. In addition, 10–15% of patients suffer from urinary incontinence [19]. In head and neck cancer patients, nerve anatomies are complex [20] and recurrent laryngeal nerve injury and mandibular nerve injury is seen in 14% of patients undergoing, respectively, thyroid surgery or neck dissection [21, 22]. Permanent paralysis is seen in 4–7% of patients [22].

**Fig. 1:**
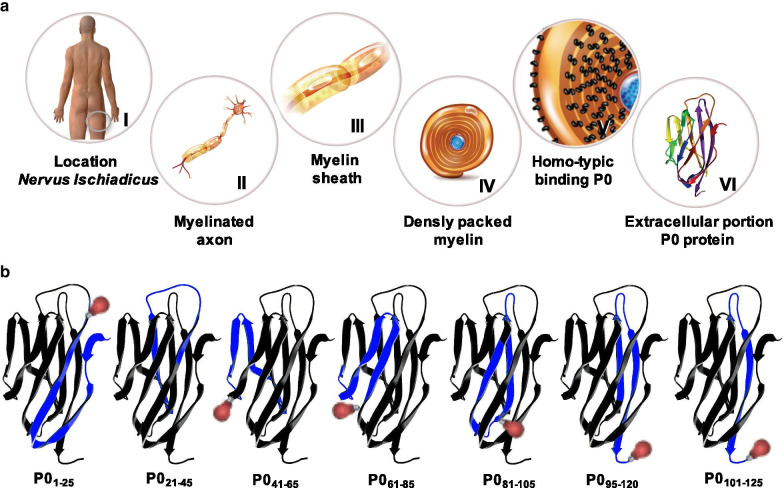
Myelin protein zero as a target for nerve imaging. **A** schematic overview of the localization of myelin protein zero (P0) in the peripheral nervous system with (I) the location of the *nervus ischiadicus* (encircled in grey), (II) myelinated axon within this nerve, (III) the myelin sheath encapsulating the axon, (IV) densely packed myelin within the myelin sheath, (V) homotypic binding of P0 within the myelin sheath (location P0 on outer membrane and between layers annotated in black) and (VI) the crystal structure of the extracellular portion of P0 (P0_ex_). **B** Peptides **P0**_**1–25**_**, P0**_**21–45**_**, P0**_**41–65**_**, P0**_**61–85**_**, P0**_**81–105**_**, P0**_**95–120**_ and **P0**_**101–125**_ derived from the crystal structure of P0_ex_ with the specific section of the amino acid sequence included in the peptide highlighted in blue and the location of Cy5 functionalization represented by a red-light bulb

Nerve-specific fluorescence imaging has been poised as a means to allow high-resolution nerve identification in real time [23]. For this application, a number of different targeting strategies have been evaluated, ranging from neuronal tracing to targeting intracellular expressed proteins in myelin sheets such as myelin basic protein [23–27]. In some cases, the nerve-specific target is unknown [27–30]. When pursuing conventional receptor-targeted molecular imaging strategies, extracellularly expressed targets are generally sought after. In that sense, myelin protein zero (MPZ, or P0), a 124-amino-acid-residues-large homotypic protein that makes up 80% of the protein content in peripheral myelin (Fig. 1A, [31]), and that is located on the outer membrane of the Schwann cells that form the myelin sheath, would most certainly be a target that is worth exploring. Uniquely, P0 is specific for the peripheral nervous system (PNS) and is not expressed in the central nervous system (CNS). In the CNS myelin sheath formation is facilitated by the adhesive properties of myelin proteolipid protein (PLP; [32]).

Building on the truncation of the extracellular portion of P0 (Fig. 1B) that was previously proposed by Makowska et al. [33] and the homotypic binding properties of P0 (e.g. intrinsic binding between P0 and P0), fluorescently labelled nerve-specific synthetic P0-derived peptides were extrapolated from the crystal structure of P0 (Fig. 1B; peptides in blue). After initial solubility, (photo)physical and in vitro evaluation (affinity and microscopic localization) a lead compound that showed the most ideal properties was selected. This fluorescent tracer was further scrutinized in three-dimensional (3D) dorsal root ganglion (DRG) cell cultures, on ex vivo nerve specimens and in vivo in mice (macroscopic localization, nerve/myelin-specificity, biodistribution). In vivo nerve visualization in a porcine model was performed as a proof of principle for real-time nerve visualization in a robot-assisted surgery setting.

## Methods

### General chemistry

All chemicals were obtained from commercial sources and used without further purification. DMF was dried over 4 Å molecular sieves. High-pressure liquid chromatography (HPLC) was performed on a Waters HPLC system (Waters Chromatography B.V., Etten-Leur, The Netherlands) using a 1525EF pump and a 2489 UV detector. For preparative HPLC, a Maisch ReproSil-Pur 120 C18-AQ 10 μM (250 mm × 20 mm) column (Dr. Maisch HPLC GmbH, Ammerbuch-Entringen, Germany) was used at a flow rate of 12 mL/min. For analytical HPLC, a Maisch ReproSil-Pur C18-AQ 5 μM (250 mm × 4.6 mm) column was used with a gradient of 0.1% trifluoroacetic acid (TFA) in H_2_O/MeCN 95:5 to 0.1% TFA in H_2_O/MeCN 5:95 in 20 min (1 mL/min). Low-resolution mass spectrometry (LRMS) was performed on a Bruker Microflex LT/SH MALDI-TOF mass spectrometer using linear mode. High-resolution mass spectrometry (HRMS) was performed on a Waters Acquity H-class UPLC (Waters, Milford, USA) using a Acquity UPLC BEH C18 1.7 μm (2.1 × 50 mm) column with a gradient of 0.1% FA in H_2_O/CH_3_CN 98:2 to 0.1% FA in H_2_O/CH_3_CN 60:40 in 1.8 min (0.6 mL/min) coupled to a high-resolution XEVO G2S-XTOF Mass Spectrometer (Waters, Milford, USA). ^1^H and ^13^C NMR were performed on a Bruker Ascend 850 (850 MHz) equipped with a CryoProbe (all from Bruker, Billerica, USA) in deuterated solvents. Crude peptides were analysed by a Waters Acquity UPLC-MS system using a Waters BEH C18 1.7 µm, 2.1 × 100 mm column, applying gradient from 5% CH_3_CN in H_2_O + 0.2% TFA to 75% CH_3_CN in 7 min.

### Peptide synthesis and Cy5 labelling

The peptides **P0**_**1–25**_**, P0**_**21–45**_**, P0**_**41–65**_**, P0**_**61–85**_**, P0**_**81–105**_**, P0**_**95–120**_ and **P0**_**101–125**_ (Fig. 1B, Additional file 1: Table SI1) were synthesized by the peptide production facility of the LUMC employing (robotic) Fmoc SPPS using preloaded Tentagel® S AC resins (Rapp Polymere GmbH, Tübingen, Germany). A pseudoproline method was employed for **P0**_**95–120**_ and **P0**_**101–125**_ [34]. Peptides **P0**_**1–25**_ (52% isolated yield)**, P0**_**41–65**_ (41% isolated yield), **P0**_**61–85**_ (22% isolated yield), and **P0**_**81–105**_ (85% isolated yield) could be synthesized in fair yields. **P0**_**95–120**_ and **P0**_**101–125**_ could only be effectively synthesized using pseudoprolines [34], resulting in a 57% and 50% yield. The synthesis of **P0**_**21–45**_ failed repeatedly, meaning this peptide was excluded from further evaluation.

Fluorescent labelling yielding **Cy5-P0**_**1–25**_**, Cy5-P0**_**41–65**_**, Cy5-P0**_**61–85**_**, Cy5-P0**_**81–105**_**, Cy5-P0**_**95–120**_ and **Cy5-P0**_**101–125**_ (see Additional file1: Scheme SI1) was achieved by dissolving 3.8 µmol of each peptide in phosphate buffer (100 mM, pH 7.4). **Cy5-Maleimide** (3.8 µmol, dissolved in DMF; See Additional file 1: Cy5-Maleimide synthesis) was added, and the reaction mixture was agitated for 2 h at room temperature followed by purification by (semi)preparative HPLC. Fluorescent conjugation yielded **Cy5-P0**_**1–25**_ (3% isolated yield), **Cy5-P0**_**41–65**_ (49% isolated yield), **Cy5-P0**_**61–85**_ (5% isolated yield), **Cy5-P0**_**81–105**_ (35% isolated yield), **Cy5-P0**_**95–120**_ (29% isolated yield), and **Cy5-P0**_**101–125**_ (59% isolated yield). The implementation of the pseudoproline method not only increased the yield when synthesizing **P0**_**101–125**_, but also increased the labelling yield (resulting in **Cy5-P0**_**101–125**_): An over sixfold increase in yield (9% isolated yield to 59% isolated yield) was seen after implementing this method; labelling of **P0**_**101–125**_ and purification of **Cy5-P0**_**101–125**_ become more efficient as this peptide could be obtained with less by-products. For more synthetic, analytical and stability details on both the peptides and fluorescent peptides, see Additional file 1: Figure SI1.

Synthesis of labelled control compounds **Cy5-P0**_**ex**_ (based on the extracellular portion of the P0 protein, Fig. 1B), **Cy5-P0**_**Ab-H60**_ (fluorescent variant of the anti P0 antibody clone H60 (**P0**_**Ab-H60**_)) and **Cy5-NP41** (based on the non-P0 staining peptide NP41 [27], and **DTPA-P0**_**101–125**_ (including its radiolabelling yielding **[**^**111**^**In]In-DTPA-P0**_**101–125**_) is described in Additional file 1: synthesis of control compounds.

### Detailed analysis of the selected lead compound Cy5-P0_101–125_

**Cy5-P0**_**101–125**_ was selected as lead compound and subjected to more detailed chemical analysis. Methods and results for assessment of the (photo)physical properties of **Cy5-P0**_**101–125**_ (i.e. serum protein binding and LogP_o/w_, chemical stability, stability at different temperatures and the molar extinction coefficient and relative quantum yield and brightness) are described in Additional file 1: chemical properties.

### Cells and animal models

P0-expressing RT4 D6P2T myelinating Schwannoma cells (ATCC® CRL-2768™; [35]) and non-P0-expressing MDAMB 468 human breast cancer cells (ATCC® HTB-132™) were grown in Dulbecco’s modified Eagle medium (Life Technologies, UK) containing penicillin, streptomycin and foetal calf serum (All BD Biosciences) at 37 °C and 5% CO_2_.

In line with their use by Whitney et al. [27], transgenic B6.Cg-Tg(Thy1-YFP)-16Jrs/J (THY-1 YFP) mice were obtained from JAX (the Jackson Laboratory) and were used for ex vivo and in vivo nerve staining and in vivo biodistribution studies (8–15 weeks old). THY-1 YFP mice express spectral variants of GFP (yellow fluorescent protein—YFP; ex 488, em 520) at high levels in motor and sensory neurons. The fluorescent signal in the nerves was used as an internal control for the staining (pattern) of the developed imaging agents. Balb/c nude mice were used as non-fluorescent control.

### Flow cytometry

Analysis of the binding affinity (K_D_) of **Cy5-P0**_**1–25**_**, Cy5-P0**_**41–65**_**, Cy5-P0**_**61–85**_**, Cy5-P0**_**81–105**_**, Cy5-P0**_**95–120**_ and **Cy5-P0**_**101–125**_ for P0 was performed using P0-expressing RT4 D6P2T cells and a previously described flow cytometric method [36]. For saturation, binding experiments were performed for each of the fluorescent peptides in a concentration range of 0–2000 nM. All measurements were taken in triplicate, and experiments were repeated at least three times per tracer. Fluorescence was measured using a FACSCanto II flow cytometry device (BD Biosciences) in the APC-A channel. The normalized geometric means were fitted with equations in the GraphPad Prism 5 software. A fluorescence-linked immunoabsorbent assay (FLISA) that was used to confirm the specificity of **Cy5-P0**_**101–125**_ for P0 is described in Additional file 1: Fluorescence-linked immunoabsorbent assay.

### Fluorescence microscopy of cells

Cells were trypsinized and seeded onto 35-mm culture dishes that contained a glass insert (MatTek co) on the day prior to the imaging experiment.

To all samples, 1 mL medium containing 1 µM **Cy5-P0**_**1–25**_, **Cy5-P0**_**21–45**_, **Cy5-P0**_**41–65**_, **Cy5-P0**_**61–85**_, **Cy5-P0**_**81–105**_, **Cy5-P0**_**95–120**_, or **Cy5-P0**_**101–125**_ was added at one hour prior to imaging (incubation at 4 °C; *N* = 3 per tracer). Peptide solutions were sonicated for 20 s prior to addition, in order to prevent aggregation of the peptides in solution. Cy5-functionalized derivatives of a P0-specific antibody (**Cy5-P0**_**Ab-H60**_), the extracellular portion of P0 (**Cy5-P0**_**ex**_) as well as a non-P0-specific peptide (**Cy5-NP-41**) and non-functionalized **Cy5-Maleimide** were used as controls (1 µM). A lysosomal (lysotracker green; 2 µL/mL, DND-26, Thermo Fisher) and nuclear stain (Hoechst 33342; 1 mg/mL, Thermo Fisher) were added as means to localize the cell nucleus and intracellular lysosomes. For blocking studies, 5 µL of a 1 mg/mL solution of non-functionalized P0_Ab_ was added to the cells one hour prior to addition of **Cy5-P0**_**101–125**_. The synthesis of the control compounds is described in the methods section of Additional file 1: Synthesis of control compounds.

In vitro and ex vivo fluorescence confocal images were acquired using a Leica SP8 WL at sequential settings and 10 × or 63 × magnification. Image analysis was performed using Leica Confocal Software (Leica Microsystems). For blocking studies, quantification of the fluorescence signal intensity (*N* = 10 for blocked and non-blocked) in the obtained images was performed using ImageJ according to previously described methods [37, 38]. Statistical evaluation was performed based on a Student’s t test.

### More detailed ex vivo and in vivo studies with lead compound Cy5-P0_101–125_

#### Culture and imaging of 3D dorsal root ganglion (DRG) explant cultures from THY-1 YFP mouse embryos

For evaluation of the staining pattern of P0, 3D DRG explant cultures were used (all *N* = 3 per peptide or control staining). Description of the methods used for ex vivo culture and imaging of 3D DRG explant cultures from THY-1 YFP mouse embryos are provided in Additional file 1: 3D culture of DRG explants. **Cy5-P0**_**Ab-H60**_, **Cy5-P0**_**ex**_ as well as **Cy5-NP-41** were used as controls.

#### Ex vivo tissue of mice

Fluorescence immunohistochemistry was performed on fresh frozen samples of the *nervus ischiadicus* that were embedded in Tissue-Tek and cut into 5 µM frozen sections. Cryo-sections were fixed in pre-cooled acetone (VWR Chemicals, 67-64-1) for 10 min and dried on air for 1 h and washed with 1 × phosphate-buffered saline (PBS) (Life Technologies, 10010-015) to remove Tissue-Tek. Slides were incubated for one hour at room temperature with 1 µM of **Cy5-P0**_**101–125**_. Sections were rinsed and dehydrated using ethanol and mounted with ProLong Gold Antifade Mountant with DAPI (Fisher, P-36931). Images were obtained using a fluorescence confocal microscope. Standard antibody-based immunohistochemistry was used as control; for details, see Additional file 1: Immunohistochemistry.

For direct ex vivo assessment of freshly excised, non-treated tissue non-fixed sections of the *nervus ischiadicus* (mouse; *N* = 3) were incubated in 1.5-mL vials (Eppendorf, Falcon) containing 1 µM **Cy5-P0**_**101–125**_ for 1 h. Confocal imaging was performed after washing with PBS. Non-incubated sections of the nerve were used as control.

#### In vivo assessment in mice

For evaluation of in vivo staining in mice, **Cy5-P0**_**101–125**_ was administered (20 μL; 5 nmol) either intravenously in the v. femoralis or directly into the nerve sheath (intraneural) of THY-1 TFP mice (*N* = 3 per injection method) or Balb/c nude mice. Injection was performed under general anaesthetics (hypnorm/dormicum/H_2_O solution (1:1:2; 5 µL/g) via intraperitoneal injection). After placement of the mouse in the microscope stand, images were collected prior and during the dissection of the *Nervus Ischiadicus*. Staining was evaluated at 1 h after injection; *N* = 3. Mice were killed via cervical dislocation before the start of the imaging session. Animals that received no tracer or that received an intravenous (v. femoralis) injection of **Cy5-NP-41** were used as control. In vivo fluorescence confocal microscopy was performed using a Zeiss 710 NLO upright confocal microscope. For collection and evaluation of the in vivo images, ZEN 2011 software was used. Furthermore, the *nervus ischiadicus* (in vivo; mouse model) and the Pudendal Nerve (excised after in vivo imaging; porcine model) were imaged using a Dino-lite handheld digital fluorescence microscope (AM4115T-DFRW for Cy5 imaging; Dino-lite Digital Microscope; λ_ex_ 620 nm, λ_em_ 650 nm). In-house developed image-processing software [39] that allowed colour coding of the fluorescence signal for improved visualization and distinction of intensity differences was used to depict the nerve-to-background ratio (NBR; ratio between relative fluorescence units in the tumour and surrounding tissue). The provided pseudo-coloured fluorescence overlay was accompanied by an intensity-based scalebar representing the NBR (fluorescence signal intensity differences represented via a colour spectrum). Confirmation of the TBR values was obtained using ImageJ software by dividing the fluorescent signal intensity in the tumour by the fluorescent signal intensity in background tissue.

#### Biodistribution of [^111^In]In-DTPA-P0_101–125_ in mice

Synthesis and radiolabelling of **DTPA-P0**_**101–125**_ with ^111^In is described in Additional file 1: synthesis of control compounds. For quantitative assessment of the biodistribution of **[**^**111**^**In]In-DTPA-P0**_**101–125**_, 10 MBq of the labelled tracer was injected intravenously (tail vein). The percentage of the injected activity per gram of tissue (%IA/g) was assessed at 2 h post-injection as previously described [40, 41]. Excretion was defined as: (MBq present in animal at 24 h post-tracer administration/injected activity) * 100%.

#### In vivo assessment in a porcine model

To evaluate whether **Cy5-P0**_**101–125**_ (100 μg, 25.6 nmol) was compatible with a real-life surgical setting, its use evaluated was in a porcine model undergoing robot-assisted surgery using a da Vinci Si or Xi system (Intuitive). Pigs (*N* = 3) were injected directly in the Pudendal nerve (intraneural administration). Using a prototype and Cy5 dedicated KARL STORZ fluorescence laparoscope [42] introduced through the assistant trocar, a similar set-up was initially applied in the clinical setting [43, 44]; fluorescence imaging of the nerve and surrounding tissues was performed at 1 h after tracer administration. Animals were maintained under Isoflurane anaesthesia for the complete duration of the surgical training and subsequent nerve imaging experiments and were euthanized before awakening from the anaesthesia. After resection, fluorescence microscopy images were made of the fresh nerve to confirm staining. Image processing was performed using in-house custom-developed software as described above.

## Results

### Peptide synthesis

As can be seen in Table 1 and Additional file 1: Scheme SI1, the various P0-peptides contain multiple amino acids that can drive self-association through ionic interactions, hydrogen bonding, hydrophobic- and van der Waals interactions [45]. These interactions may play a role in peptides **Cy5-P0**_**41–65**_, **Cy5-P0**_**61–85**_, and **Cy5-P0**_**81–105**_ forming particles in solution. Although sonication could be used to (partially) overcome this aggregation, **Cy5-P0**_**95–120**_ and **Cy5-P0**_**101–125**_ show a 2- to 29-fold higher solubility which promotes their use.

**Table 1:** Fluorescently labelled P0 peptides, sequences and outcome synthesis

Peptide	Amino acid sequence	Negative/positive charges	Solubility in H_2_O (µM)	K_D_ in nM
Cy5-P0_1–25_	H-IVVYTDREVHGAVGSQVTLHC(Cy5)SFWS-NH_2_	4+/4−	12	> 1000
Cy5-P0_41–65_	Ac-PEGGRDAISIFHYAKGQPYIDEVGTC(Cy5)-NH_2_	3+/7−	45	> 1000
Cy5-P0_61–85_	Ac-DEVGTFKERIQWVGDPRWKDGSIVIC(Cy5)-NH_2_	5+/8−	6	> 1000
Cy5-P0_81–105_	Ac-GSIVIHNLDYSDNGTFTC(Cy5)DVKNPPD-NH_2_	2+/6−	68	> 1000
Cy5-P0_95–120_	Ac-TFT*A*DVKNPPDIVG**KT**SQ**VT**LYVFEKC(Cy5)-NH_2_^a^	4+/5−	150	> 1000
Cy5-P0_101–125_	Ac-KNPPDIVG**KT**SQ**VT**LYVFEKVPTRYC(Cy5)-NH_2_^a^	4+/6−	172	105 ± 17

^a^Italic alanine residue replacing the cysteine from native P0, bolded and underlined residues were implemented via the above-mentioned pseudoproline method [34], underlined cysteines were non-native residues added to the C-terminus

### In vitro analysis

Quantitative assessment of the binding affinity based on saturation binding experiments revealed a nanomolar (or submicromolar) binding constant (K_D_) of 105 ± 17 nM for **Cy5-P0**_**101–125**_ (Additional file 1: Figure SI3A/B, Table 1). For the five other Cy5-P0 peptides within the matrix K_D_ values of > 1000 nM was found (Table 1).

**Fig. 2:**
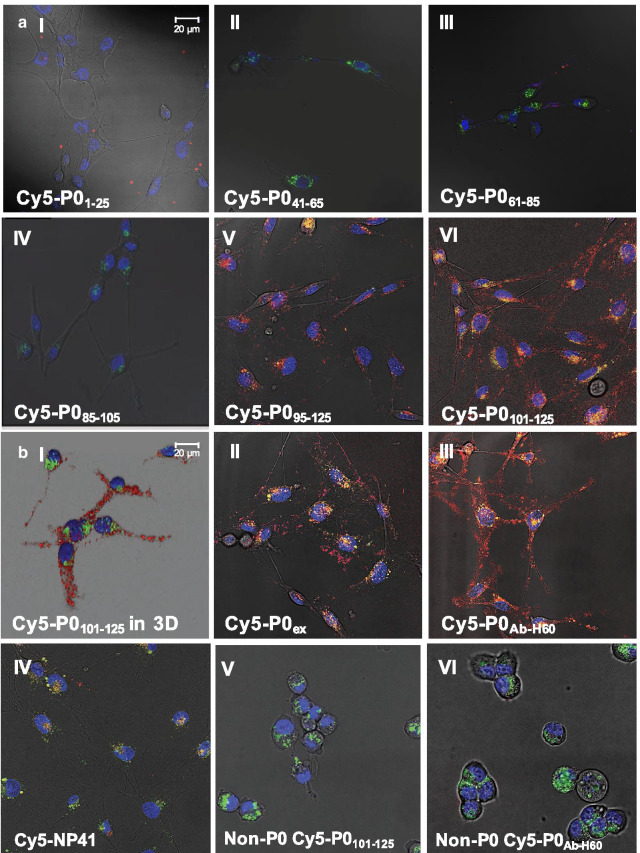
Localization of binding of P0 peptides to myelinating Schwannoma cells. Fluorescence confocal images of RT4 D6P2T Schwannoma cells after incubation with **A** C-terminally Cy5-labelled P0_1–25_(**Cy5-P0**_**1–25**_; I), P0_41–65_ (**Cy5-P0**_**41–65**_; II), P0_61–85_ (**Cy5-P0**_**61–85**_; III), P0_85–105_(**Cy5-P0**_**185–105**_; IV), P0_95–120_ (**Cy5-P0**_**95–125**_; V) or P0_101–125_ (**Cy5-P0**_**101–125**_; VI). **B** Fluorescence confocal images of RT4 D6P2T Schwannoma cells after incubation with **Cy5-P0**_**95–120**_ represented in 3D (I), and the extracellular portion of P0 (**Cy5-P0**_**ex**_; II), Cy5-labelled anti-P0 antibody clone H60 (**Cy5-P0**_**Ab-H60**_; III), a non-P0-specific peptide (**Cy5-NP41**; IV). Staining of non-P0-expressing MDAMB 468 cells with **Cy5-P0**_**101–125**_ (V), **Cy5-P0**_**Ab**_ (VI) and Cy5-Maleimide (CI) were used to show specificity for P0. Blocking experiments showed a clear decrease in the mean fluorescence intensity for **Cy5-P0**_**101–125**_ after pre-incubation with P0_Ab_ (II). Quantification of fluorescence intensity with and without blocking (III; **Cy5-P0**_**101–125**_ in red, blocked conditions in black) further underlined specificity (*p* = 0.001). In all confocal images, Cy5 is represented in red, nuclear staining (Hoechst) in blue and lysosomes (lysotracker green) in green

Fluorescence confocal microscopy of P0-expressing Schwannoma cells revealed clear differences in staining patterns between the tracers (Fig. 2A). While no clear staining was seen for **Cy5-P0**_**1–25**_**, Cy5-P0**_**41–65**_**, Cy5-P0**_**61–85**_ or **Cy5-P0**_**85–105**_ (Fig. 2A I–IV), staining of the Schwannoma cells with **Cy5-P0**_**95–120**_**,** and especially **Cy5-P0**_**101–125**_ resulted in a densely spotted pattern on both the cell body and the cell outgrowths (Fig. 2A V and VI; Cy5 in red). **Cy5-P0**_**101–125**_ was superior in both the degree of staining and signal intensity, which helped finalize its selection as lead compound. 3D assessment of the cell specimens confirmed that localization of staining of **Cy5-P0**_**101–125**_ was distinctly different to that of lysosomes, confirming extracellular, instead of intracellular, staining (Fig. 2B I and Additional file 1: Figure SI4). The location of **Cy5-P0**_**101–125**_ accumulation was in agreement with staining of an anti-P0 antibody (**Cy5-anti-P0**_**Ab-H60**_; Fig. 2B II) as well as staining with the extracellular portion of P0 (**Cy5-P0**_**ex**_; Fig. 2B III). P0-related staining was not seen with the non-P0-specific control peptide (**Cy5-NP41**; Fig. 2B IV; [27]) nor was a similar staining seen in P0-negative cells stained with **Cy5-P0**_**101–125**_ (Fig. 2B V), **Cy5-anti-P0**_**Ab-H60**_ (Fig. 2B VI) or when the non-functionalized Cy5-Maleimide dye was applied (Fig. 2C I).

Blocking experiments revealed the specificity of **Cy5-P0**_**101–125**_ for P0 (Fig. 2CII and III); Quantification of the fluorescence intensity of **Cy5-P0**_**101–125**_ with (Fig. 2CIII, in red) or without addition of P0_Ab_ (in black) showed that only 13.5 ± 7.1% of Cy5-related fluorescence that can be contributed to binding of **Cy5-P0**_**101–125**_ remained after blocking (*p* = 0.001). A customized FLISA set-up further underlined the specificity of **Cy5-P0**_**101–125**_ for P0 by confirming the binding to the extracellular portion of P0 (P0_ex_) for both **Cy5-P0**_**101–125**_ and **Cy5-P0**_**Ab-H60**_. No binding was observed for the non-P0-specific control **Cy5-NP41** (Additional file 1: Figure SI3).

### More detailed studies with lead compound Cy5-P0_101–125_

#### Chemical analysis

Chemical analysis of **Cy5-P0**_**101–125**_ revealed that this tracer was 99% stable after incubation in serum for 24 h at 37 °C and > 99% stable at temperatures > 0 °C for at least 4 h (Additional file 1: Figure SI2). Additional chemical- and photophysical features of **Cy5-P0**_**101–125**_ are presented in Additional file 1: Table SI2.

#### Imaging of 3D dorsal root ganglion (DRG) explant cultures from THY-1 YFP mouse embryos

**Fig. 3:**
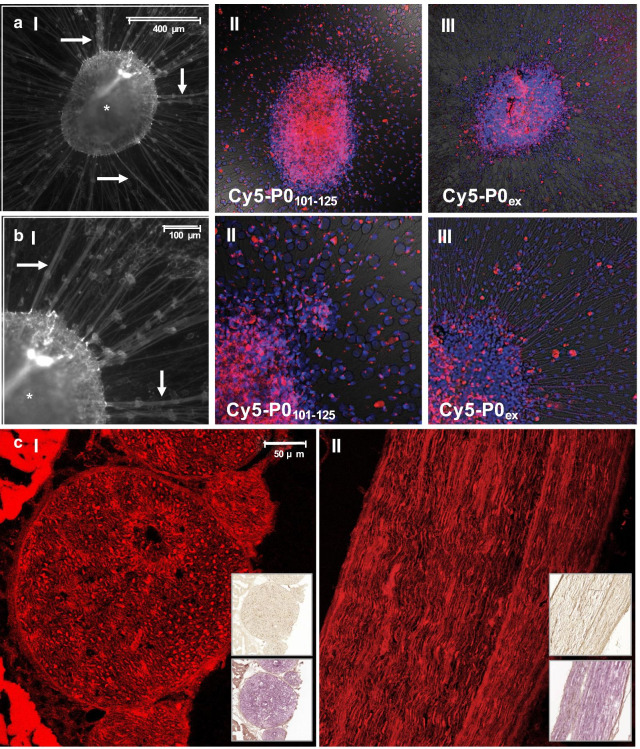
Staining of 3D DRG explant cultures with Cy5-P0_101–125_ and Cy5-P0_101–125_ fluorescence immunohistochemistry of a murine *nervus ischiadicus*. **A** brightfield image of a 3D DRG explant (*) with axonal outgrowths (white arrow) after staining with (II) **Cy5-P0**_**101–125**_ or (III) **Cy5-P0**_**ex**_. **B** Zoom-in with focus on the axonal outgrowths and fluorescence confocal imaging of DRG explants after staining with (II) **Cy5-P0**_**101–125**_ or (III) **Cy5-P0**_**ex**_. **C** Fluorescence immunohistochemistry of the *nervus ischiadicus* of THY1-YFP mice in (I) transverse orientation and (II) sagittal orientation after staining with **Cy5-P0**_**101–125.**_ The top insert shows standard anti-body-based standard immunohistochemistry and the bottom insert H&E staining of a concurrent/adjacent tissue section

3D cultures based on DRG explants obtained from THY-1 YFP mouse embryos provided an intermediate step between in vitro and in vivo evaluation (Fig. 3A, B). These 3D cultures contained a centre ganglion (*) and axonal outgrowths (white arrow) and are known as well-established neuronal cultures for drug discovery for neuronal neuropathies [46]. Incubation with **Cy5-P0**_**101–125**_ resulted in a spotted staining pattern of cells residing along the course of the developed axonal outgrowths as well as in the DRG explant itself (Fig. 3AII and AIII). Again, staining with **Cy5-P0**_**ex**_ (Fig. 3AIII and BIII) confirmed the findings.

#### Ex vivo assessment of murine nerve tissue

Immunohistochemical assessment of concurrent fresh-frozen sections of the *nervus ischiadicus* of THY1-YFP mice revealed a clear overlap between the location of P0 between staining obtained after incubation with **Cy5-P0**_**101–125**_ (fluorescence immunohistochemistry; Fig. 3C) and an anti-P0 antibody (standard immunohistochemistry; insert Fig. 3C).

**Fig. 4:**
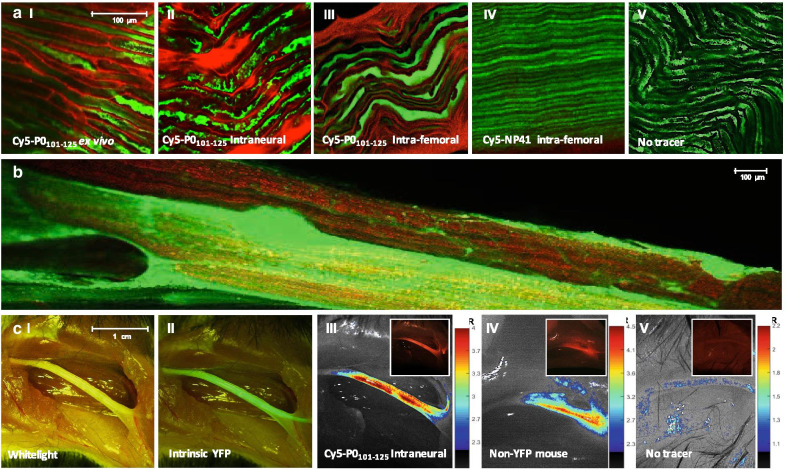
Ex vivo and in vivo imaging of the *nervus ischiadicus* of mice. **A** 63 × magnification of the *nervus ischiadicus* after (I) ex vivo incubation, (II) intraneural administration or (III) intravenous (v. femoralis) administration of **Cy5-P0**_**101–125**_. (IV) Fluorescence confocal image of the *nervus ischiadicus* **F** after intravenous (v. femoralis) administration of **Cy5-NP41** or (V) when no tracer was applied. **B** In vivo fluorescence confocal image of a large field of view of the *nervus ischiadicus* of a THY-1 YFP at 1 h after intravenous (v. femoralis) administration of **Cy5-P0**_**101–125**_. Cy5 in red and intrinsic YFP located in the axons of THY-1 YFP mice in green. **C** In vivo Dinolight microscopy images showing (I) a whitelight image of the *nervus ischiadicus* and (II) the intrinsic YFP signal in a THY-1 YFP mouse. Image processing after illumination of the *nervus ischiadicus* after intraneural administration of **Cy5-P0**_**101–125**_ in (III) a THY-1 YFP mouse, (IV) a non-YFP Balb/c nude mouse and (V) when no tracer was applied. Inserts show unprocessed fluorescence image. Scalebar represents the nerve-to-background ratio (NBR)

More detailed microscopic assessment of viable (non-frozen, non-pretreated) samples of the *nervus ischiadicus* that were incubated ex vivo with **Cy5-P0**_**101–125**_ revealed an identical wavy staining pattern (Fig. 4AI; in red). Here, the intrinsic YFP signal within the axons of the THY-1 YFP mice provided an extra confirmation that staining of **Cy5-P0**_**101–125**_ co-localized with the myelin sheath surrounding the axons**.**

#### In vivo assessment in mice

To reduce the dose and staining beyond the area of interest, in vivo administration intraneural (Fig. 4AII) and intravenous (v. femoralis; Fig. 4AIII and B) administration were applied. Both tracer administration routes provided identical results compared to ex vivo incubation with **Cy5-P0**_**101–125**_. Similar to assessment in vitro (Fig. 2), the non-P0-specific peptide **CY5-NP41** (Fig. 4AIV) did not provide staining corresponding to the location of myelin after intravenous administration (v. femoralis). Intraoperative fluorescence confocal microscopy after administration of **Cy5-P0**_**101–125**_ allowed clear visualization of the *nervus ischiadicus* based on the emitted fluorescence signal (Fig. 4B).

**Fig. 5:**
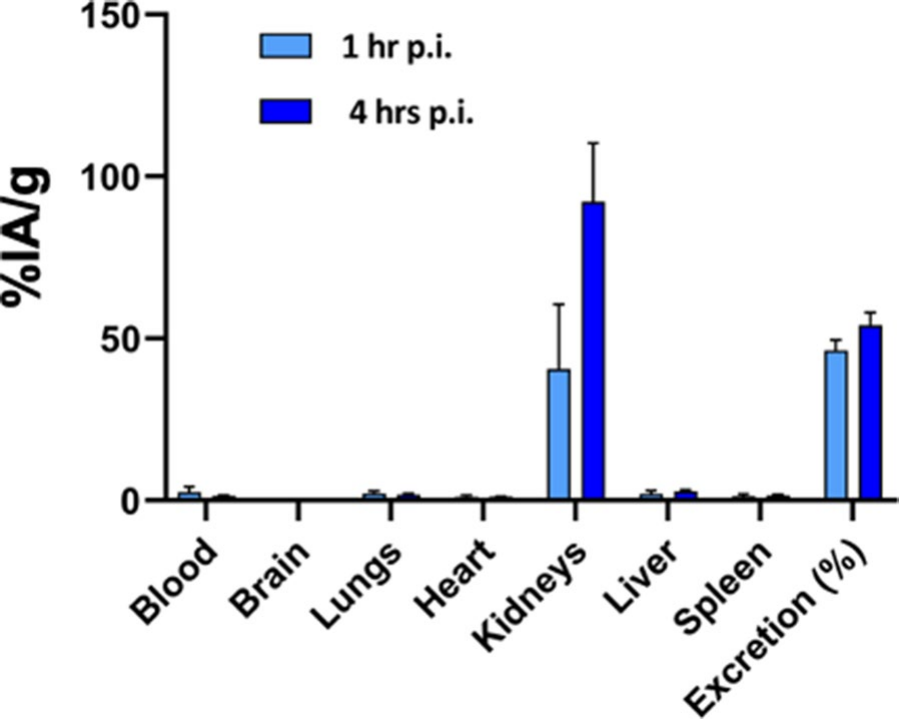
In vivo biodistribution. Quantitative in vivo biodistribution and excretion of **[**^**111**^**In]In-DTPA-P0**_**101–125**_ at 1 (light blue) and 4 h (dark blue) post-intravenous tracer administration (tail vein). Uptake per organ presented as percentage of the injected activity per gram of tissue (%IA/g). Excretion was defined as: (MBq present in animal at 24 h post-tracer administration/injected activity) * 100%

Macroscopic in vivo assessment of the *nervous ischiadicus* (whitelight image; Fig. 5C) resulted in a mean NBR of 6.0 ± 2.2 after administration of **Cy5-P0**_**101–125**_ (Fig. 4CIII). Comparable results in non-YFP mice (Fig. 5CIV; mean NBR: 3.7 ± 1.0) help exclude the possibility of spectral overlap between YFP and Cy5.

#### Biodistribution (mouse)

Radiolabelling of **DTPA-P0**_**101–125**_ with ^111^In (yielding **[**^**111**^**In]In-DTPA-P0**_**101–125**_) allowed quantitative assessment of the biodistribution at 1 and 4 h after intravenous tracer administration (Fig. 5). At both time points (1 and 4 h) renal clearance, low overall tissue uptake (in %IA/g) and a substantial level of excretion were seen. Most importantly, uptake in the CNS was neglectable (0.12 ± 0.03%IA/g at 4 h p.i.).

#### In vivo assessment in a porcine model

**Fig. 6:**
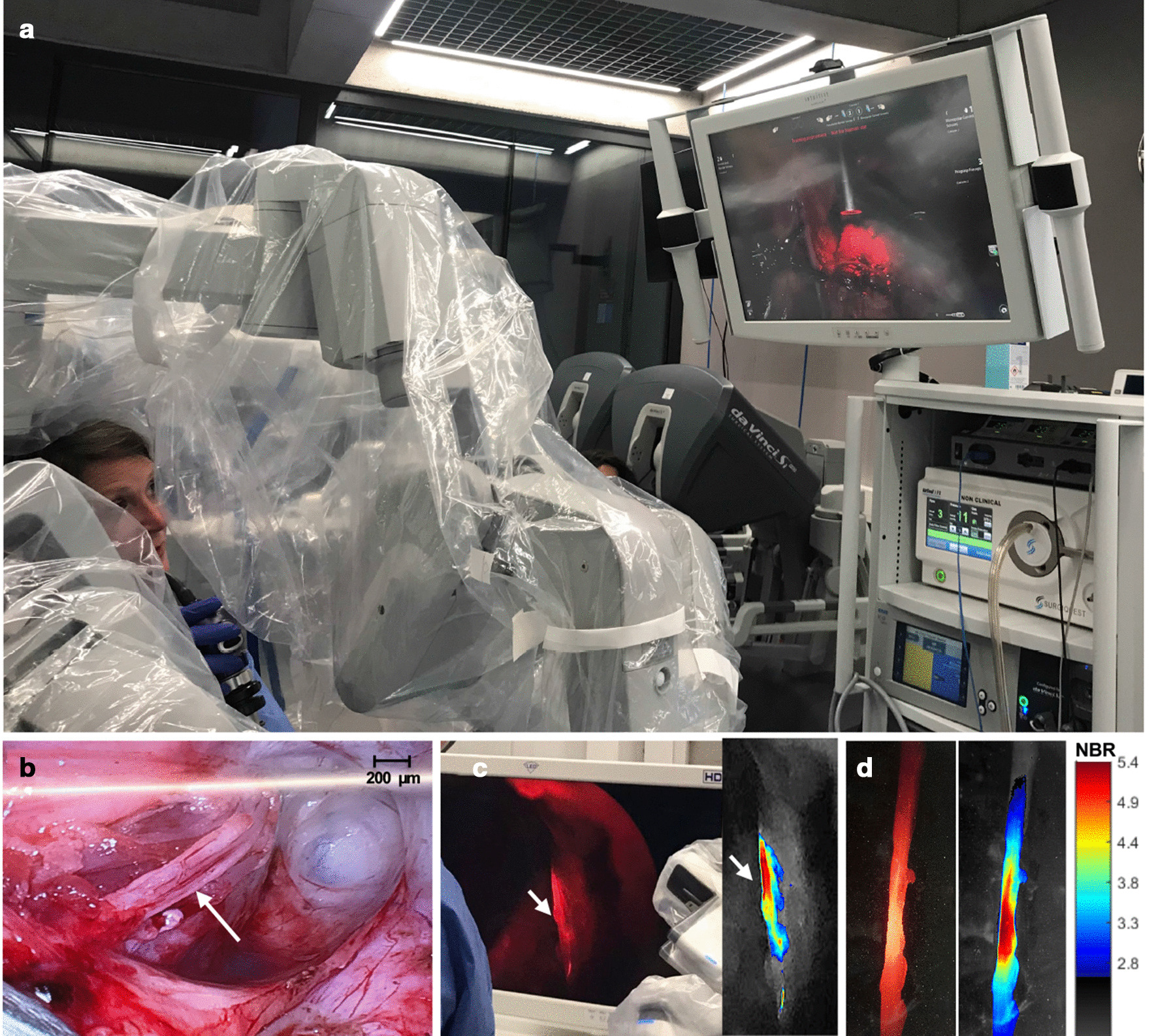
Translation of Cy5-P0_101–125_ into large animal models using clinical grade imaging modalities. **A** Surgical set-up showing the Da Vinci surgical robot and the use of a clinical grade STORZ fluorescence laparoscope dedicated for Cy5 imaging [42]. **B** Brightfield image of the pudendal nerve (white arrow). **C** In vivo fluorescence imaging of the pudendal nerve after intraneural administration of **Cy5-P0**_**101–125**_ as visible on the screen of the imaging set-up. Insert showing a colour-coded processed image of the fluorescence in the nerve. **D** Ex vivo fluorescence image of the excised nerve (left image) with corresponding colour-coded image-processing (right image) and scale bar depicting the corresponding nerve-to-background ratio (NBR)

The compatibility of **Cy5-P0**_**101–125**_ with clinical grade imaging modalities applied during robot-assisted minimally invasive surgery was evaluated in a porcine model (Fig. 6). Here, the use of a dedicated Cy5 fluorescence laparoscope (KARL STORZ; [42]) allowed in vivo visualization of the pudendal nerve (Fig. 6B, C; white arrow). Image processing based on colour coding of the fluorescence signal helped assess differences in fluorescence intensity along the nerve (Fig. 6C insert). Back-table imaging of the excised specimen confirmed the fluorescence of the nerve (Fig. 6D).

## Discussion

By using the P0-derived synthetic peptide **Cy5-P0**_**101–125**_, we were able to explore the homotypic P0 protein as molecular target that is widely expressed in myelin in the PNS. Specific binding was demonstrated in vitro, in 3D DRG nerve cultures, ex vivo, as well as in vivo.

Truncation of the homotypical P0-protein into peptides yielded **Cy5-P0**_**101–125**_ as lead compound. In vitro and in vivo studies indicate that this compound is able to provide nerve-specific staining with nanomolar affinity, which is in the same range as reported affinities for other fluorescently labelled targeted tracers [36, 47, 48]. Specificity of **Cy5-P0**_**101–125**_ for P0 was shown both in vitro (Fig. 2 and Additional file 1: Figure SI3) and in vivo (Fig. 4). An approximate 90% decrease in fluorescence intensity after pre-incubation with a P0-specific antibody was seen (Fig. 2CIII), while no staining was observed for the non-P0-specific control (**Cy5-NP41**) and the free dye (Cy5-Maleimide). As the Cy5-Maleimide dye variant was used for functionalization of both P0_101–125_ and NP41, these results also exclude a targeting effect of the dye itself. This was corroborated by a markedly different staining pattern using free dye alone [30]. An additional beneficial factor for **Cy5-P0**_**101–125**_ is that the production is scalable and can be done at reasonably low cost. Moreover, the peptide benefits from the superior pharmacokinetics that have been claimed for peptides over proteins such as antibodies [49, 50].

In line with P0 expression, biodistribution studies performed with the radiolabelled analogue **[**^**111**^**In]In-DTPA-P0**_**101–125**_ helped rule out accumulation of **P0**_**101–125**_ (MW = 3024 Da) in the CNS. Obviously, the chance of toxic side effects is also impacted by dosing and uptake in non-target organs. In nuclear medicine, disease-specific tracers are therefore applied using a micro-dosing regimen (< 100 µg/patient). Although the ability of using fluorescence at micro-dosing levels looks promising [51], this topic remains a subject of debate [52], and many studies still use high dosing regimens to realize in vivo functionalization of molecular targets [16, 53–55]. Local tracer deposition (an image-guided surgery concept that has proven valuable in e.g. lymphatic mapping and during occult lesion localization) limits dosing to 100 µg/patient [12, 51]. Recent experimental studies underscore that local deposition may also be valid when targeting peripheral nerves within a certain surgical anatomy [24, 37]. Substantiated by previous reports [28] Fig. 4 AIII and B indicate that local tracer administration is feasible for nerve imaging applications.

While most image-guided surgery studies promote the use of near-infrared (NIR) Cy7 analogues [53, 54, 56], far-red Cy5-labels are also increasingly being applied in clinical trials [39, 57–59]. In fact, fluorescence-guided surgery trials have been reported for the full fluorescent light spectrum [60]. Uniquely, in a head-to-head comparison, Cy5 analogues even were shown to outperform Cy7-analogies in terms of signal intensity [42] and impact on tracer kinetics [36]. Moreover, Cy5 analogues, both a free dye and conjugated to a peptide, have shown to have a high fluorescence brightness in the presence of human serum albumin (Additional file 1: Table SI2; [36, 41, 61]). These factors combined with the fact that most groups are creating tumour-receptor-targeted tracers using NIR Cy7 analogues support the future implementation of multi-wavelength imaging applications, a concept that is gaining traction in the clinic [60].

Although intraneural injection provides a perfect proof of principle in both mice and pigs (Figs. 4AII, C and Additional file 1: Figure SI6), administration into a blood vessel near the target organ, such as the femoral vein (Fig. 4A), still provides further optimization before it can be used in a human setting. Hence, the exact route for local tracer administration in large animals, minimization of the dose and the specificity of targeting remain the subject of ongoing studies. Another potential limitation of the approach presented is that myelinization of nerves in the PNS may vary, where sensory nerves are highly myelinated (and conductive), the level of myelination decreases substantially in autonomous nerves. Despite the fact that myelin is one of the most widely explored targets for nerve imaging, it is therefore not clear if myelin-specific tracers will help address all surgical nerve imaging demands.

## Conclusion

By truncating the P0 protein, we have been able to successfully create a nerve-specific fluorescent tracer that is able to specifically stain P0/myelin expression in the PNS.

## Supplementary Information

**Additional file 1.** Errors regarding SI table/scheme references were corrected.

## Data Availability

The datasets used and/or analysed during the current study are available from the corresponding author on reasonable request.

## Abbreviations

MPZ, or P0: Myelin protein zero
PNS: Peripheral nervous system
CNS: Central nervous system
PLP: Myelin proteolipid protein
3D: Three-dimensional
DRG: Dorsal root ganglion
HPLC: High-pressure liquid chromatography
LRMS: Low-resolution mass spectrometry
SI: Supporting information
PBS: Phosphate-buffered saline
FLISA: Fluorescence-linked immunoabsorbent assay
NIR: Near-infrared
