# Supplementary material for: Intraoperative visualization of nerves using a myelin protein-zero specific fluorescent tracer
Source: EJNMMI Res. 2021 May 29;11:50. doi: 10.1186/s13550-021-00792-9 (PMC8164657; doi:10.1186/s13550-021-00792-9)
Supplement: Supplementary file 1 — Additional file 1. Errors regarding SI table/scheme references were corrected. [file 13550_2021_792_MOESM1_ESM.pdf]

Supporting information

Intraoperative visualization of nerves using a myelin protein-zero specific fluorescent tracer.

Authors: Tessa Buckle[1*], Albertus.W. Hensbergen[1], Danny M. van Willigen[1], Frank Bosse[2], Kevin Bauwens[3], Rob C.M. Pelger[4], Fijs W.B. van Leeuwen[1,3*]

1) Interventional Molecular Imaging Laboratory, Department of Radiology, Leiden University Medical Center, Leiden, the Netherlands; 2) Neurologische Klinik, Heinrich-Heine University Dusseldorf, Dusseldorf, Germany; 3) ORSI Academy, Melle, Belgium; 4) Department of Urology, Leiden University Medical Center, Leiden, the Netherlands

Material and methods

Peptide synthesis workup and yields

After deprotection and cleavage of all peptides from the resin using TFA/H_2O/ethanedithiol 18:1:1 for 3 h, and at last triethyl silane for 10 min, the crude peptides were precipitated in cold MTBE/hexanes followed by washing with MTBE/hexanes and subsequently drying and lyophilisation. Analysis of the crude peptide was performed using a Waters Acquity LCMS with a Waters BEH C18 1.7 µm, 2.1x100 mm column, applying gradient from 5% CH_3CN in H_2O + 0.2% TFA to 75% CH_3CN in 7 min. The resulting purities were used for the yield calculation. The peptides eluted between 2.9 and 3.3 min. **$P0_{1–25}$** ($[M+H]^+$ m/z = calcd. 2819.2, found 2819.1; isolated yield: 52%), **$P0_{41–65}$** ($[M+H]^+$ m/z = calcd. 2866.2, found 2866.2; isolated yield: 41%), **$P0_{61–85}$** ($[M+H]^+$ m/z = calcd. 3075.5, found 3075.3; isolated yield: 22%), **$P0_{81–105}$** ($[M+H]^+$ m/z = calcd. 2763.0, found 2763.9; isolated yield: 56%), **$P0_{95–120}$** ($[M+H]^+$ m/z = calcd. 3043.5,

found 3042.9; isolated yield: 57%), **P0**$_{101–125}$ ($[M+H]^+$ m/z = calcd. 3025.5, found 3025.2; isolated yield: 14%), and **P0**$_{101–125}$ using pseudoproline method ($[M+H]^+$ m/z = calcd. 3025.5, found 3025.2; isolated yield: 50%).

Table SI1. P0 peptide, sequences, calculated isoelectric point (pI) and net charge at pH 7.0.

**Peptide**	**Amino acid sequence**	**pI*** **	**Net charge (pH 7.0)*** **
**P0**$_{1–25}$	H-IVVYTDREVHGAVGSRVTLHCSFWS-NH$_2$	7.3	0.1
**P0**$_{21–45}$*	Ac-CSFWSSEWVSDDISFTWRYQPEGGR-NH$_2$*	4.1	-2.0
**P0**$_{41–65}$	Ac-PEGGRDAISIFHYAKGQPYIDEVGTC-NH$_2$	4.5	-2.0
**P0**$_{61–85}$	Ac-DEVGTFKERIQWVGDPRWKDGSIVIC-NH$_2$	4.6	-1.0
**P0**$_{81–105}$	Ac-GSIVIHNLDYSDNGTFTCDVKNPPD-NH$_2$	3.9	-3.0
**P0**$_{95–120}$	Ac-TFTADVKNPPDIVG**KT**SQ**VT**LYVFEKC-NH$_2$ **	6.4	0.0
**P0**$_{101–125}$	Ac-KNPPDIVGKTSQVTLYVFEKVPTRYC-NH$_2$	9.7	2.0
**P0**$_{101–125}$	Ac-KNPPDIVG**KT**SQ**VT**LYVFEKVPTRYC-NH$_2$ **	9.7	2.0

* synthesis unsuccessful, ** highlighted alanine residue replacing the cysteine from native P0, bolded and underlined residues were implemented via the abovementioned pseudoproline method (1), underlined cysteines were non-native residues added to the C-terminus. ***

Cy5-Maleimide synthesis

Cy5-COOH (28.5 mg, 37.4 µmol); synthesised following previously published procedures (2) was dissolved in dry DMSO (800 µL), followed by the addition of PyBOP (39.4 mg, 75.8 µmol), aminoethyl maleimide trifluoroacetate (9.3 mg, 36.6 µmol) and *N*-methyl morpholine (16.4 µL, 149.6 µmol). The mixture was stirred for 90 min at r.t. Afterwards, the crude product was purified using preparative HPLC. Lyophilisation yielded **Cy5-Maleimide** as a blue, fluffy solid

(8.1 mg, 51%) with a purity of 98%. HRMS calculated for $C_{41}H_{48}N_4O_{12}S_3^{2-}$ ([M-2H]2-), 442.1221, found 442.1250 (Figure SI1A). ^{1}H NMR (850 MHz, DMSO) δ 8.34 (td, J = 13.2, 3.5 Hz, 2H), 7.87 (t, J = 6.1 Hz, 1H), 7.80 (dd, J = 5.3, 1.2 Hz, 2H), 7.62 (ddd, J = 8.1, 4.3, 1.4 Hz, 2H), 7.36 (d, J = 8.3 Hz, 1H), 7.30 (d, J = 8.3 Hz, 1H), 6.99 (s, 2H), 6.59 (t, J = 12.3 Hz, 1H), 6.39 (d, J = 13.9 Hz, 1H), 6.30 (d, J = 13.7 Hz, 1H), 4.07 (p, J = 7.4 Hz, 4H), 3.43 – 3.40 (m, 2H), 3.17 (dd, J = 11.7, 6.0 Hz, 2H), 2.53 – 2.51 (m, 2H), 1.96 (t, J = 7.4 Hz, 2H), 1.80 – 1.71 (m, 4H), 1.68 (s, 12H), 1.65 (dd, J = 15.1, 7.6 Hz, 2H), 1.52 – 1.45 (m, 2H), 1.31 (dt, J = 15.3, 7.8 Hz, 2H; (Figure SI1B). ^{13}C NMR (214 MHz, DMSO) δ 173.08, 172.72, 172.12, 171.05, 154.34, 154.01, 145.34, 145.17, 141.99, 141.85, 140.55, 140.45, 134.52, 126.04, 125.93, 119.89, 119.85, 110.26, 110.01, 103.75, 103.33, 50.56, 48.89, 48.80, 43.54, 43.36, 37.29, 36.70, 35.05, 33.47, 31.30, 27.12, 27.06, 26.64, 25.86, 25.67, 25.60, 24.70, 24.18, 22.46 (Figure SI1C).

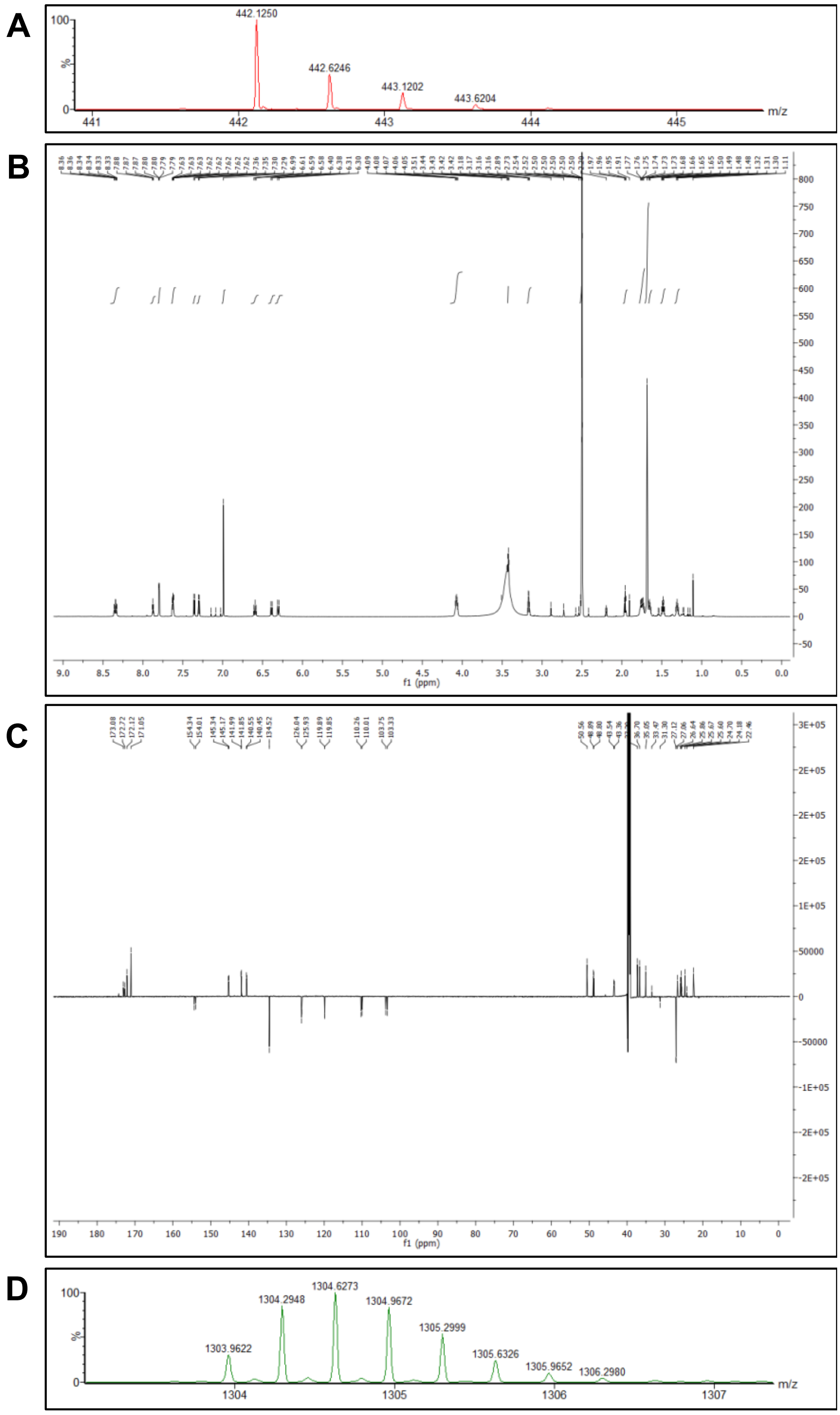

Figure SI 1. Characterization of compounds. A) HRMS spectrum of **Cy5-Maleimide.** B) ^{1}H NMR spectrum of Cy5-Maleimide. C) ^{13}C APT spectrum of **Cy5-Maleimide.** D) HRMS spectrum of **Cy5-P0**$_{\mathbf{101\text{-}125}}$.

Fluorescent peptide synthesis workup and yields

Preparative HPLC was performed using a gradient of 5-95% CH_3CN in H_2O (0.1% TFA) in 40 min. The product-containing fractions were combined and lyophilized yielding the fluorescent peptides as fluffy, light blue solids: **Cy5-P0$_{1–25}$** ([M+6H]4+ m/z = calcd. 3706.7, found 3706.8; yield: 3%), **Cy5-P0$_{41–65}$** ([M+6H]4+ m/z = calcd. 3753.6, found 3754.1; isolated yield: 49%), **Cy5-P0$_{61–85}$** ([M+7H]5+ m/z = calcd. 3964.8, found 3964.0; isolated yield: 5%), **Cy5-P0$_{81–105}$** ([M+5H]3+ m/z = calcd. 3650.5, found 3651.6; isolated yield: 35%), **Cy5-P0$_{95–120}$** ([M+3H]+ m/z = calcd. 3927.8, found 3928.4; isolated yield: 29%), **Cy5-P0$_{101–125}$** HRMS calculated for $C_{179}H_{272}N_{39}O_{51}S_4^{3+}$ ([M+5H]3+), 1304.6274, found 1304.6273 (Fig SI1D); isolated yield: 9%), and **Cy5-P0$_{101–125}$** using pseudoproline method ([M+3H]+ m/z = calcd. 3909.9, found 3910.7; isolated yield: 59%; Figure SI1D). **P0$_{21–45}$** was excluded from labelling due to insufficient yield/failed synthesis of the peptide.

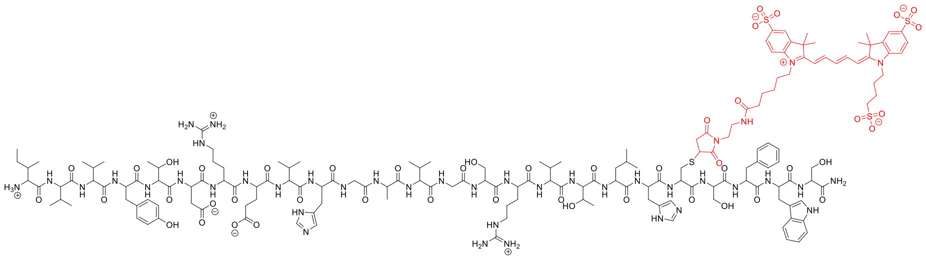

P0$_{1-25}$

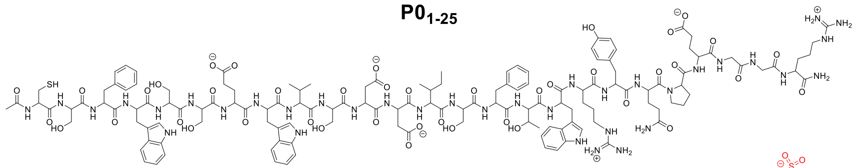

P0$_{21-45}$

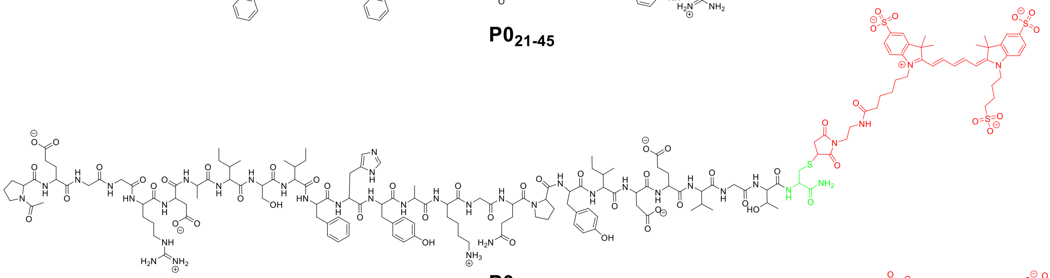

P0$_{41-65}$

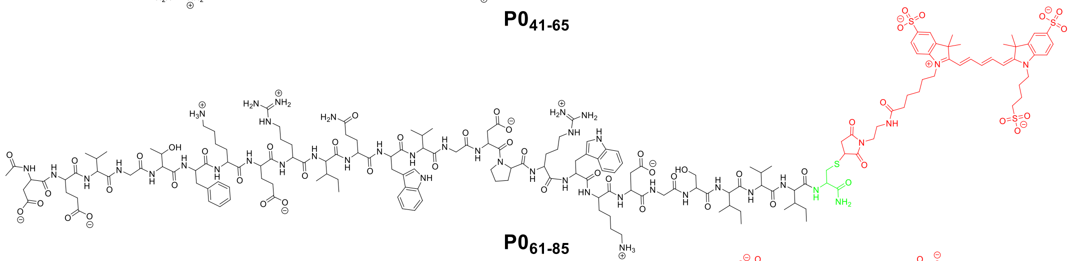

P0$_{61-85}$

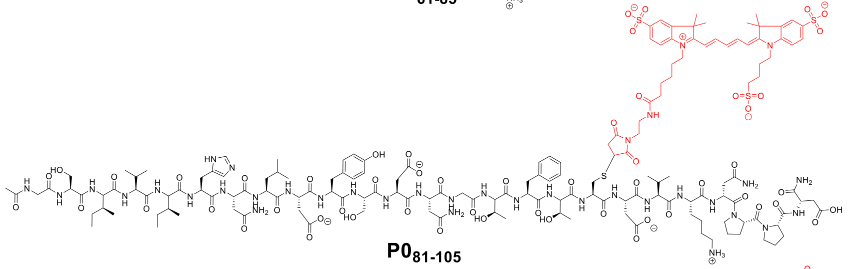

P0$_{81-105}$

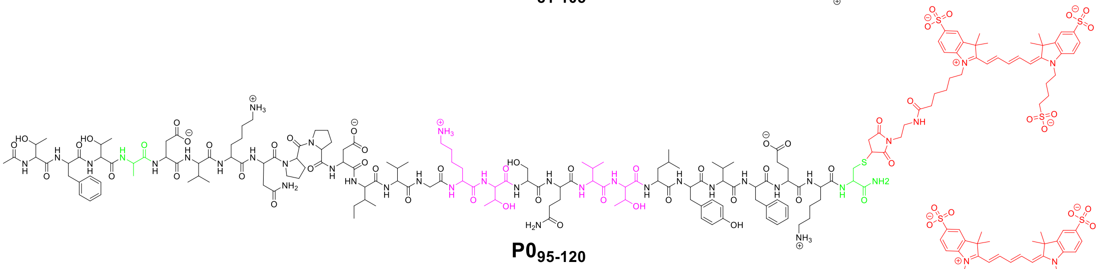

P0$_{95-120}$

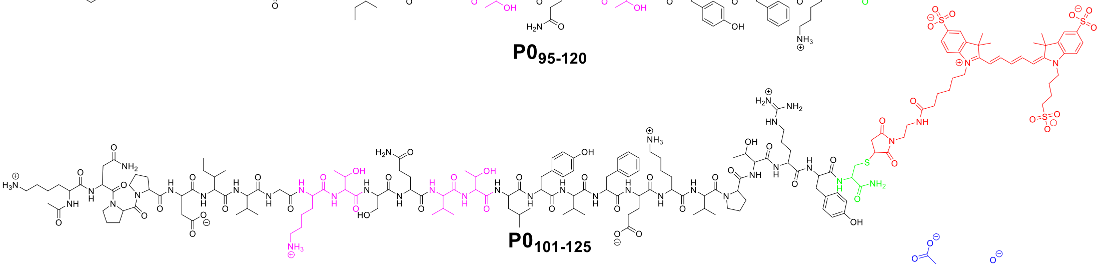

P0$_{101-125}$

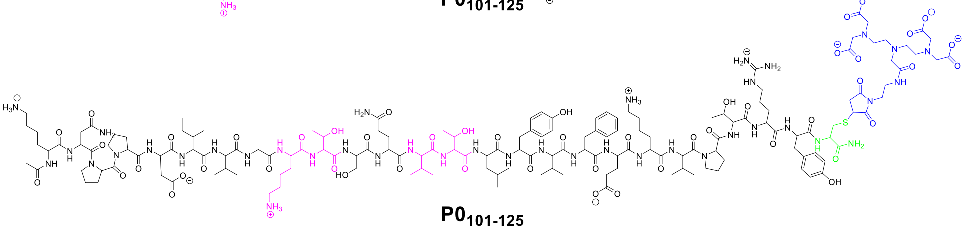

P0$_{101-125}$

Scheme SI1. Chemical structures P0-peptides. Chemical structure (including charges) of the fluorescently labelled peptides **Cy5-P0$_{1–25}$**, **Cy5- P0$_{21–45}$**, **Cy5-P0$_{41–65}$**, **Cy5-P0$_{61–85}$**, **Cy5-P0$_{81–105}$**, **Cy5-P0$_{95–120}$** and **Cy5-P0$_{101–125}$**. **Cy5-P0$_{101–125}$**. Conjugation of DTPA to P0$_{101-125}$ yielded **DTPA-P0$_{101–125}$**. Cy5-Maleimide is depicted in red, amino acids non-natively present in the sequence in green and locations where pseudo prolines were used in the synthesis in purple. DTPA is presented in blue.

Synthesis of control compounds

P0$_{ex}$ protein

BL21/DE3 bacteria that were transformed with a S11IEG3 plasmid containing a GST-tag linked to the open reading frame of the extracellular portion of P0 (P0$_{ex}$), were cultured to obtain the complete extracellular domain after lysation of the bacteria (3). (Transfected bacteria stored at -80 ^{0}C were thawed and re-suspended in PBS (4 mL; pH 8) + 1mM DTT + protease inhibitor before being sonicated on ice (10 x 10 sec). 1% Triton X-100 and 100 mg lysozyme were added to the bacteria suspension in PBS (1 mL). The suspension was then incubated at 4 °C for 1–2 h, where after DNase (100 µl of a 100 units/µL solution) was added. After 15–30 min incubation the suspension was centrifuged at 8,000 rpm for 30 min at 4 °C. The pellet was re-suspended in a mixture of Tris (8 mL; pH 8; 50 mM), DTT (1 mM), and protease inhibitor. Finally, inclusion bodies containing P0$_{ex}$ were obtained after additional centrifugation of the suspension (30 min at 8,000 rpm; 4 °C).

P0$_{ex}$ was extracted from the inclusion bodies by re-suspending the obtained pellet in a solution (5–7 mL) of urea (5 M), Tris (50 mM; pH 8; containing DTT (1 mM) and protease inhibitor). This suspension was incubated on ice for 2–3 h before centrifugation at 8,000 rpm for 30 min at 4 °C. The supernatant was transferred onto a pre-soaked 70 mL dialysis cassette and diluted with PBS/Tris (10 mM; pH 8) containing DTT (1 mM) and protease inhibitor to a

volume of 60–70 mL and dialysed in 20% glycerol in PBS (pH 8; 4 L) for 2–3 h, followed by overnight dualization in PBS/Tris (10 mM; pH 8) containing DTT (0.5 mM).

The GST-tag present on $P0_{ex}$ was used to isolate $P0_{ex}$ from the supernatant. To do so, $P0_{ex}$-containing supernatant was centrifuged at 4,000 rpm for 20 min before being transferred to tubes containing GST-coated beads. These beads were washed several times with PBS (pH 8) containing protease inhibitor. The GST-tag on $P0_{ex}$ was left to bind to the beads overnight at 4 °C. The beads were then centrifuged (20 min; 1,500 rpm) and the pellet was washed three times with PBS (20 mL; pH 8.0), followed by a washing step in Tris (3 mL; 50mM; pH 8). To extract $P0_{ex}$ from the beads, reduced glutathione (100 mM) in Tris (50 mM; pH 8) was added and incubated overnight at 4 °C. The mixture was then centrifuged at 1,500 rpm for 10 min, where after the supernatant containing $P0_{ex}$ was obtained, yielding approximately 900 µg of $P0_{ex}$, determined using a standard bicinchoninic acid assay (4).

Cy5-P0

$P0_{ex}$ (100 µg, 1.33 nmol) was washed using a 10K MWCO Amicon filter (Merck, New Jersey, USA) and phosphate buffer (0.1 M, pH 8.4). After collection of the concentrate, phosphate buffer (0.1 M, pH 8.4, 100 µL) and **Cy5-OSu** (5.9 µL, 16.00 nmol) were added. The mixture was stirred for 3 h where after the unbound fluorophore was separated by centrifuging using a new 10K MWCO Amicon filter. When a colourless filtrate was observed, the labelled protein concentrate was collected, and the degree of labelling was estimated to be 1.45 dye/protein using absorption spectrometry (Nanodrop).

Cy5-P0$_{AB\text{-}H60}$

Anti P0 antibody clone H60 (**P0$_{Ab\text{-}H60}$**; 200 µg, 1.33 nmol, SCBT, Texas, USA) was washed using a 10K MWCO Amicon filter (Merck, New Jersey, USA) and 0.1M phosphate buffer pH 8.4. After collection of the concentrate, phosphate buffer (0.1 M, pH 8.4; 200 µL) and **Cy5-OSu** (1.3 µL, 6.67 nmol) were added. The mixture was stirred for 135 min where after the unbound fluorophore was separated by centrifuging using a new 10K MWCO Amicon filter. When a colourless filtrate was observed, the labelled protein concentrate was collected, and the degree of labelling was estimated to be 1.26 dye/protein using absorption spectrometry (Nanodrop).

Cy5-NP41

The crude peptide (9.8 mg, 4,5 µmol) was dissolved in 0.1M phosphate buffer pH 7.75 (2 mL, purged with N_2) and to this **Cy5-Maleimide** (8.0 mg, 9.00 µmol) dissolved in DMF. The solution was stirred overnight at r.t. After purification by preparative HPLC, **Cy5-NP41** was obtained as a blue solid. LRMS calculated $[M+3H]^+$ for $C_{115}H_{170}N_{29}O_{39}S_4^+$ 2711.0, found 2711.0.

DTPA-P0$_{101–125}$

P0$_{101–125}$ (2.0 mg, 1.1 µmol) was dissolved in phosphate buffer (pH = 7.0, 100 mM; 500 µL). DTPA-maleimide (1.1 mg, 2.1 µmol, Chematech, Dijon, France) was then added. The resulting mixture was stirred at room temperature (r.t.) for 20 h before adding TFA (60 µL). The crude product was purified by means of prep-HPLC, obtaining the title compound as a white solid (1.8 mg, 31%). LRMS calculated $[M+H]^+$ for $C_{158}H_{249}N_{40}O_{50}S^+$ 3541.0, found 3543.2. Radiolabelling of **DTPA-P0$_{101\text{-}125}$** with ^{111}In yielded **[^{111}In]In-DTPA-P0$_{101\text{-}125}$** (used for

assessment biodistribution and uptake in CNS) and was carried out and assessed as previously described (5, 6).

Chemical properties

The solubility of **Cy5-P0$_{1–25}$**, **Cy5-P0$_{41–65}$**, **$_{Cy5}$-P0$_{61–85}$**, **Cy5-P0$_{81–105}$**, **Cy5-P0$_{95–120}$** and **Cy5-P0$_{101–125}$** was determined by dissolving the labelled peptides a saturated solution in H_2O at r.t., centrifugation and measuring absorption of the supernatant using an Ultrospec 3000 UV/Vis spectrometer (Pharmacia Biotech).

Serum protein binding and LogP$_{o/w}$

Serum protein binding and lipophilicity in octanol/water of **Cy5-P0$_{101-125}$** and non-functionalized **Cy5** were determined as previously described (7).

Chemical stability

The assessment of the stability in serum of **Cy5-P0$_{101-125}$** and non-functionalized **Cy5** at 24 h at 37°C was carried out as described previously (5). The stability towards glutathione of **Cy5-P0$_{101-125}$** was determined as described by van der Wal et al (8), with the following deviations: compound stock was prepared in H_2O, samples were incubated in a water bath at 37°C, analysis was performed using a Waters 1525EF equipped with a 2489 UV/Vis detector, using a SunFire C18 Column, 100Å, 3.5 µm, 4.6 mm X 150 mm column and a gradient from 5:95 CH_3CN:H_2O + 0.1% TFA to 5:95 H_2O: CH_3CN + 0.1% TFA in 24 min.

Stability at different temperatures

A stock solution of either non-functionalized **Cy5** or **Cy5-P0$_{101-125}$** (10 µM in PBS) was diluted 1:1 in PBS to create a concentration range between 10–0.6 µM. The absorbance and fluorescence intensities of each sample were measured (t=0 h) prior to storing the samples at different temperatures (i.e., -20, 0, 20 and 37 °C). After t = 4 h, the absorbance and fluorescence intensities of each sample was measured. The relative intensities (compared to t=0) of each concentration were averaged to obtain n = 6 per temperature setting.

Molar extinction coefficient, relative quantum yield and brightness

The molar extinction coefficient, relative quantum yield and brightness of **Cy5-P0$_{101-125}$** were determined following published procedures (7) using a compound stock in H_2O and a concentration range of 0.25-7.50 µM.

Table SI2. Chemical properties and brightness of Cy5-P0$_{101–125}$ and non-functionalized Cy5.

	**Cy5-P0$_{101–125}$**	**Cy5**
**Absorption/<br>Emission in PBS (nm)**	650/671	648/664*
**Absorption/<br>Emission in HSA (nm)**	651/668	649/666
**Lipophilicity (o/w)**	-1.39 ± 0.09	-1,42 ± 0,00
**Serum protein binding**	89% ± 2%	6% ± 5%*
**Stability in serum**	99%	98%
**Stability towards glutathione**	92%	99%*
**Molar extinction coefficient in PBS**	117.000	242.000*
**Molar extinction coefficient in HSA**	121.146	N.D.

**Relative quantum yield in PBS**	23%	22%*
**Relative quantum yield in HSA**	22%	21%
**Brightness in PBS**	26.910	53.240*
**Brightness in HSA**	26.652	N.D.

*: data collected or calculated from previous reports by Spa et al (2).

Fluorescence-linked immunoabsorbent assay

Lumitrac 600 96 wells plates were coated with P0-ex (10 μg/well) and incubated overnight at 4 ˚C. The coated wells were then blocked with 200 µl casein (2.5 g/100 mL) per well and incubated for two hours at room temperature. Plates were washed two times with PBS containing 0.05% Tween 20 (Life Technologies Inc.) For saturation binding experiments **Cy5-P0$_{101–125}$** (32 µM) was added to the wells in a range of 0 – 4000 nM. **Cy5-P0$_{Ab-H60}$** (three different concentrations used; 300 nM, 66 nM or 17 nM) and **Cy5-NP41** (2000 nM) were used as controls. After a two-hour incubation of the peptides or controls the wells were washed three times with 0.05% Tween 20 in PBS and absorbance of the Cy5 conjugates was measured at 680 nm (excitation 630 nm) using a SpectraMax microplate reader containing a cut-off filter at 665 nm.

3D culture of dorsal root ganglion (DRG) explants from THY-1 YFP mouse embryos

Glass coverslips were pre-coated overnight with poly-L-lysine (20 μg/mL). On the day of DRG dissection, coverslips were further coated with laminin 40 μg/mL in DMEM/F12 and incubated for 2 hours at 37 °C. Hereafter, the coverslips were rinsed with DMEM/F12 and transferred to 24 well plates containing 500 μl of culture medium. Culture medium (DMEM/F12 with Glutamax; 10mL) containing 100 μl N_2 supplement (100x stock; Sigma 17502), 20ng/mL NGF (20 μl, 10 μg/mL stock) and 100 μl 1x PenStrep.

Embryos were dissected at embryonic day 13 (E13) in L15 (Leibovitz) medium *via* a previously described method (9, 10) and transferred into HBSS solution immediately after cervical dislocation of a pregnant THY-1 YFP mouse. Attached nerve roots and other unwanted tissue were removed from the collected DRGs, which were then transferred into DMEM/F12 culture medium, onto the laminin-coated glass coverslips. Axonal outgrowths from the DRGs were allowed to grow for 48 hours (at 37 °C and 5% CO2), prior to incubation with the different imaging agents and subsequent imaging.

Staining of Dorsal Root Ganglion explants

Laminin-coated glass coverslips with DRG explants were placed on top of the glass insert of a 35 mm culture dish before addition of the imaging agent to the medium. For ex vivo tissue experiments excised sections of the *Nervus Ischiadicus* were incubated in 1.5 mL vials (Eppendorf, Falcon).

To all samples 1 mL medium was added, containing 1 µM **Cy5-P0**$_{\mathbf{101–125}}$ at one hour prior to imaging (incubation at 4 ^{o}C). Peptide solutions were sonicated for 20 seconds prior to addition, in order to prevent aggregation of the peptides in solution. A lysosomal (lysotracker green; 2 µL/mL, DND-26, Thermo Fisher) and nuclear stain (Hoechst 33342; 1 mg/mL, Thermo Fisher) was added as means to localize the cell nucleus and intracellular lysosomes. **Cy5-P0**$_{\mathbf{Ab\text{-}H60}}$, **Cy5-P0**$_{\mathbf{ex}}$ as well as **Cy5-NP-41** were used as controls.

Immunohistochemistry

For antibody-based stainings cryo-sections were fixed in pre-cooled acetone (VWR Chemicals, 67-64-1) for 10 minutes and dried on air for one hour. Then, sections were washed with 1x Phosphate-buffered saline (PBS; Life Technologies, 10010-015) to remove Tissue-Tek.

Endogenous peroxidase was inhibited with 0.3% H_2O_2/PBS for 20 minutes, then sections were rinsed and incubated overnight Chicken-anti-Myelin Protein Zero antibody (Abcam, ab134439) in a humidified chamber at r.t. After rinsing, sections were incubated with biotinylated Goat-anti-Chicken secondary antibody Abcam, ab6876) for 45 minutes in a humidified chamber at r.t.. Rinsed sections were incubated with ABC reagents (Vector Labs ®, ZF1011) in a humidified chamber at r.t. for 45 minutes. After addition of Then peroxidase of 3,3'-Diaminobenzidine (DAB; DAKO, 10060875), sections were counterstained with Haematoxylin (Sigma Aldrich, GHS132-1L) and mounted with DePeX mounting medium (EMS, 13514). Images were obtained using a Pannoramic MIDI digital slide scanner (3DHISTECH) and then processed using CaseViewer (3DHISTECH).

For hematoxylin and eosin staining Sections were washed with 1x Phosphate-buffered saline (PBS) (Life Technologies, 10010-015), stained for 10 minutes with Gils Hematoxylin (Sigma Aldrich, GHS132-1L) and rinsed for 5 minutes with distilled water. Sections were dipped 12 times in 0.5% Eosin and rinsed with distilled. Then, sections were dehydrated using 50%, 70%, 95% EtOH and Xylene (Sigma Aldrich, 534056-4L) and were then mounted with DePeX (EMS, 13514). Images were taken using a Pannoramic MIDI digital slide scanner (3DHISTECH) and then processed using CaseViewer (3DHISTECH).

Results

Detailed analysis of the selected lead compound Cy5-P0$_{101\text{-}125}$

Serum protein binding and lipophilicity

The serum protein binding of the lead compound **Cy5-P0$_{101\text{-}125}$** (89 ± 2%) was 15-fold higher when compared to the free fluorophore **Cy5** (6% ± 5% (2)) despite the statistically

nonsignificant difference in lipophilicity ($LogP_{o/w}$ = -1.39 ± 0.09 for **Cy5-P0$_{101-125}$**, LogP = -1.42 ± 0.00 for **Cy5.**

Stability towards glutathione and in serum

Cy5-P0$_{101-125}$ showed only 8% degradation when incubated with glutathione for 6 hr at 37 °C, which was in line with previous results for **Cy5** (1% degradation; (2)). Furthermore, both **Cy5-P0$_{101-125}$** as well as **Cy5** are virtually not susceptible to degradation by serum proteins (98–99% stable after 24 h).

Stability at different temperatures

The stability of **Cy5-P0$_{101-125}$** over a 4-hour timespan is most likely not influenced by temperature (Figure SI2). The decrease in absorbance (66 ± 8% remaining) and fluorescence signal (80 ± 10% remaining) for Cy5 after 4 hr at -20 °C is most likely caused by stacking of the cyanine dye, a feature we reported on previously (2). It is likely that the presence of the peptide inhibited the aggregation of **Cy5-P0$_{101-125}$** (95 ± 9% remaining absorbance, 91 ± 5% remaining fluorescence after 4 hr at -20 °C).

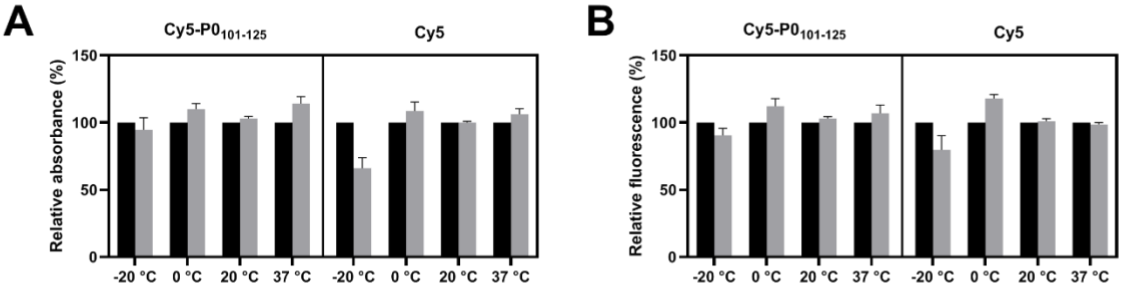

Figure SI2. Stability of Cy5-P0$_{101-125}$ and non-functionalized Cy5. Stability measurements showing A) Absorbance and B) fluorescence of **Cy5-P0$_{101-125}$** and unfunctionalized Cy5 were performed at t=0, whereafter samples were placed at either -20, 0, 20, or 37 ^{0}C and tested again after 4 hours.

Brightness comparison between Cy5 and the lead compound ***Cy5-P0$_{101\text{-}125}$***

Cy5-P0$_{101\text{-}125}$ and **Cy5** have a similar relative quantum yield (Φ_F = 23% for **Cy5-P0$_{101\text{-}125}$**, Φ_F = 22% for **Cy5**(2); (Table SI2)). The differences in molar extinction coefficients (ε = 117.000 $M^{-1} \cdot cm^{-1}$ for **Cy5-P0$_{101\text{-}125}$**, ε = 242.000 $M^{-1} \cdot cm^{-1}$ for **Cy5**) mean the brightness of **Cy5-P0$_{101\text{-}125}$** (26.910 $M^{-1} \cdot cm^{-1}$ in PBS and 26.652 $M^{-1} \cdot cm^{-1}$ in HSA) is almost two-fold lower when compared to **Cy5** (53.240 $M^{-1} \cdot cm^{-1}$), (2); Table SI2).

Evaluation of binding specificity using a fluorescence-linked immunoabsorbent assay (FLISA)

Using a custom made FLISA setup, a comparison was made between the degree of P0 binding by **Cy5-P0$_{101–125}$**, **Cy5-P0$_{Ab}$** and the non-P0 specific **Cy5-NP41** (Figure SI13) to P0$_{ex}$. Herein the highest level of binding to the P0$_{ex}$ coated on the wells was seen for **Cy5-P0$_{Ab}$** (115 AU). Clear binding was also seen for **Cy5-P0$_{101–125}$**, although with a two-fold lower readout (45 AU). No P0-related binding was seen for **Cy5-NP41** as for this compound the measured fluorescence after removal of the unbound compound was comparable to the binding to uncoated wells. This level was also comparable to the read-out found for **Cy5-P0$_{101–125}$** and **Cy5-P0$_{Ab}$**, when added to uncoated wells.

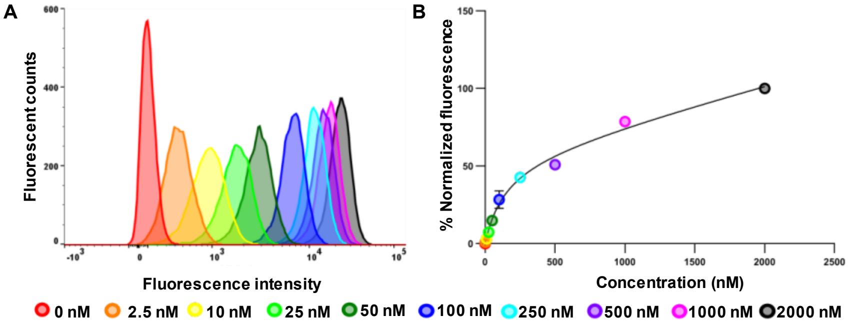

C

	Binding to P0$_{ex}$ (AU)	Uncoated control (AU)
Cy5-P0$_{101\text{-}125}$	45	2
Cy5-P0$_{Ab\text{-}H60}$	115	0
Cy5-NP41	5	-1

Figure SI3. Saturation binding and FLISA set-up for additional assessment specificity of Cy5-P0$_{101–125}$. A) Flow cytometry histogram showing an increase in fluorescence intensity with increasing concentration of **Cy5-P0$_{101–}$**

$_{125}$ (0-2000nM; N=3) with color coding of the concentrations and B) the saturation binding curve derived from these results. C) Quantified FLISA results for binding of **Cy5- P0$_{101-125}$**, **Cy5-P0$_{Ab-H60}$** and **Cy5-NP41** to either P0ex precoated wells or uncoated controls.

*3D analysis fluorescence confocal of cells incubated with **Cy5-P0$_{101-125}$***

3D analysis of Schwannoma cells incubated with **Cy5-P0$_{101-125}$** allowed detailed assessment of the localization of the P0-related staining (Figure SI4; in red) compared to intracellular organelles wherein staining of **Cy5-P0$_{101-125}$** was shown not to co-localize with nuclear and/or lysosomal staining (nucleus in blue and lysosomes in green). This was further substantiated by assessment of stained cells in different directions and placement in the z-plane; While the overview from the top of the sample (Figure SI4A I) revealed presence of all three signals, localization of the Cy5 signal (Figure SI4 AI; center cross) was deemed not to be intracellular based on images obtained in axial (II) and coronal (III) orientation. The other way round, when focusing on the intracellular staining (Figure SI4 BI; center cross) nuclear and lysosomal staining were clearly represented in axial (II) and coronal (III) assessment, without Cy5 being present.

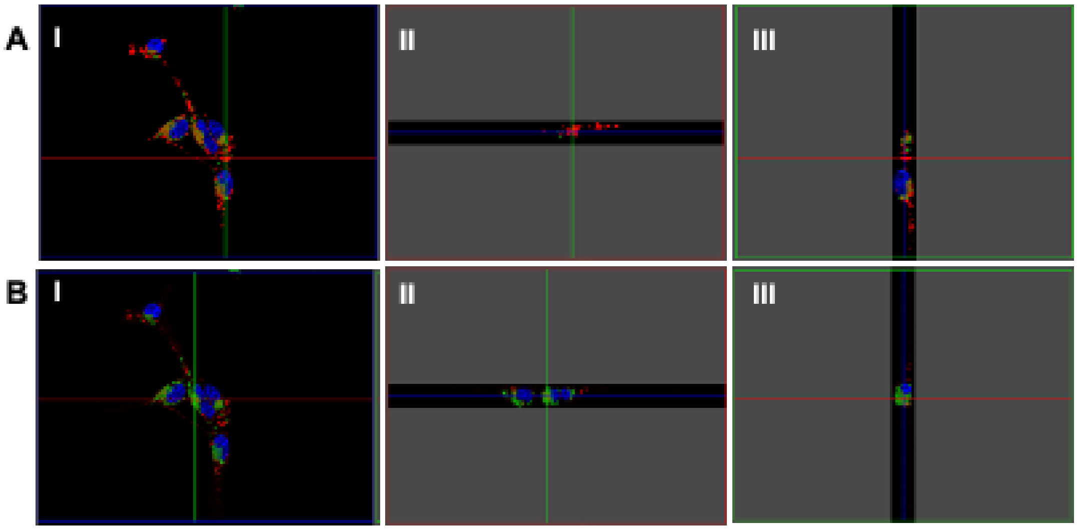

Figure SI 4. 3D analysis fluorescence confocal of cells incubated with Cy5-P0$_{101-125}$. 3D fluorescence confocal image of RT4 Schwannoma cells incubated with Cy5-P0$_{101-125}$ with A) focus on Cy5 staining (red) and B) focus on lysosomal staining (green) presented as I) top view, II) slice in axial direction, III) slide in coronal direction. Location of each cross section is highlighted by either a green (axial) or red (coronal) cross section line.

References

1. Haack T, Mutter M. Serine derived oxazolidines as secondary structure disrupting, solubilizing building blocks in peptide synthesis. Tetrahedron Letters. 1992;33(12):1589-92.

2. Spa S.J. HAW, van der Wal S., Kuil J., van Leeuwen F.W.B. The influence of systematic structure alterations on the photo-physical properties and conjugation characteristics of asymmetric cyanine 5 dyes. Dyes and Pigments. 2018;152:19-28.

3. Hasse B, Bosse F, Hanenberg H, Müller HW. Peripheral myelin protein 22 kDa and protein zero: domain specific trans-interactions. Mol Cell Neurosci. 2004;27(4):370-8.

4. Walker JM. The Bicinchoninic Acid (BCA) Assay for Protein Quantitation. In: Walker JM, editor. The Protein Protocols Handbook. Totowa, NJ: Humana Press; 1996. p. 11-4.

5. Bunschoten A, van Willigen DM, Buckle T, van den Berg NS, Welling MM, Spa SJ, et al. Tailoring Fluorescent Dyes To Optimize a Hybrid RGD-Tracer. Bioconjug Chem. 2016;27(5):1253-8.

6. Hensbergen AW, van Willigen DM, Welling MM, van der Wijk FA, de Korne CM, van Oosterom MN, et al. Click Chemistry in the Design and Production of Hybrid Tracers. ACS Omega. 2019;4(7):12438-48.

7. Hensbergen AW, Buckle T, van Willigen DM, Schottelius M, Welling MM, van der Wijk FA, et al. Hybrid Tracers Based on Cyanine Backbones Targeting Prostate-Specific Membrane Antigen: Tuning Pharmacokinetic Properties and Exploring Dye-Protein Interaction. J Nucl Med. 2020;61(2):234-41.

8. van der Wal S KJ, Valentijn ARPM, van Leeuwen FWB. Synthesis and systematic evaluation of symmetrtric sulfonated centrally Ce C bonded cyanine near-infrared dyes for protein labeling. Dyes and Pigments. 2016;132:7-19.

9. Niclou SP, Franssen EH, Ehlert EM, Taniguchi M, Verhaagen J. Meningeal cell-derived semaphorin 3A inhibits neurite outgrowth. Mol Cell Neurosci. 2003;24(4):902-12.

10. Sleigh JN, Weir GA, Schiavo G. A simple, step-by-step dissection protocol for the rapid isolation of mouse dorsal root ganglia. BMC Res Notes. 2016;9:82.
